# Food insecurity in older female mice affects food consumption, coping behaviors, and memory

**DOI:** 10.1371/journal.pone.0250585

**Published:** 2021-04-29

**Authors:** Samantha M. Estacio, Madalyn M. Thursby, Noel C. Simms, Vanessa A. Orozco, Jessica P. Wu, Alyssa A. Miawotoe, Whitney W. Worth, Claire B. Capeloto, Kyla Yamashita, Kayla R. Tewahade, Katherine B. Saxton

**Affiliations:** 1 Department of Psychology, Santa Clara University, Santa Clara, California, United States of America; 2 Public Health Program, Santa Clara University, Santa Clara University, Santa Clara, California, United States of America; 3 Department of Biology, Santa Clara University, Santa Clara University, Santa Clara, California, United States of America; Technion Israel Institute of Technology, ISRAEL

## Abstract

Food insecurity correlates with poor physical and mental health in older individuals, but has not been studied in a laboratory animal model. This explorative study developed a laboratory mouse model for analyzing the impact of food insecurity on food consumption, stress coping mechanisms, exploratory behavior, and memory. 18-month-old CD-1 female mice were assigned to either the food insecurity exposure condition (31 mice, 8 cages) or the control condition (34 mice, 8 cages) by cage. Over four weeks, the mice that were exposed to food insecurity received varied, unpredictable portions of their baseline food consumption (50%, 75%, 125%, 150% of baseline) for four days, followed by *ad libitum* access for three days, to approximate the inconsistent access to food observed in households experiencing food insecurity. Behavioral tasks were conducted before and after food insecurity exposure. Mice in the food insecurity exposure condition ate less compared to control mice during food insecurity (two-way ANOVA: group x time interaction: F_7,93_ = 10.95, P < 0.01) but ate more when given access to high fat food (two-way ANOVA, group x time interaction: F_1,14_ = 11.14, P < 0.01). Mice exposed to food insecurity increased active escaping behaviors in the forced swim test (repeated measures two-way ANOVA, group x time interaction: F_1,63_ = 5.40, P = 0.023). Exploratory behaviors were unaffected by food insecurity. Mice exposed to food insecurity showed a reduction in memory (repeated measures two-way ANOVA, group x time interaction: F_1,61_ = 4.81, P = 0.037). These results suggest that exposure to food insecurity is associated with differences in food consumption patterns, active coping mechanisms, and memory. The behavioral changes associated with food insecurity may inform research on food insecurity’s impact on health in elderly humans.

## Introduction

### Societal context

Food insecurity is one of the most pressing public health concerns in the United States, affecting 11.8% of households in 2017 [1], and increasing dramatically during the COVID-19 pandemic [2]. The U.S. Department of Agriculture (USDA) defines food insecurity as “a lack of consistent access to enough food for an active healthy life” [1]. Unrelated to voluntary dieting, food insecurity occurs when there is insufficient or uncertain availability of, and access to, nutritional foods due to social and economic problems [3]. Food insecurity varies according to various risk factors, including, but not limited to, income, employment, neighborhood, race, and ethnicity [3]. Although government food assistance programs, such as the Supplemental Nutrition Assistance Program (SNAP), have been shown to reduce food-related hardships, monthly benefits last for 4 or more weeks in only 13.6% of households using food programs, leaving families who are experiencing food insecurity with limited resources for food budgets by the end of the month [4, 5]. Tight budgets and unexpected expenses may force individuals to decide between food or other necessities, such as rent, utilities, and medical bills; this can lead to unpredictable changes in food availability throughout the month.

### Food choice availability

The limited food choices often available to individuals experiencing food insecurity, including high sugar and high fat foods, contribute to negative health outcomes [6]. Food insecurity may increase the risk of poor physical health, including obesity, diabetes, hypertension, and other chronic diseases [7–10]. Unhealthy food, such as fast food and soda, has a lower cost per calorie than more nutritional options [11, 12]. Therefore, the inaccessibility and unaffordability of healthy food choices may increase the risk of obesity [13] and diabetes [14] for individuals experiencing food insecurity. Research has also shown that chronic stress can increase cravings for foods that are high in sugar and fat [15].

### Health impacts in humans

Food insecurity is also linked to a variety of mental health concerns such as stress, depression, anxiety, and suicidality [9]. Experiencing food insecurity is associated with poorer mental health in a dose-response fashion, where more extreme food insecurity is correlated to worse mental health outcomes [16]. Additionally, lower socioeconomic status, which is highly associated with food insecurity, was found to be associated with worse physical and mental health due to higher vulnerability to multiple risk factors and the chronic stress of living in poverty [6].

### Food insecurity in vulnerable populations

While food insecurity has been shown to negatively impact individuals during a variety of different developmental periods, [see 6, 17], it is especially important to consider the impact of exposure to food insecurity on elderly individuals as it has been linked to depression, anxiety, and limited ability to perform daily living activities [18], as well as poorer physical health [19]. The impact of food insecurity on elderly individuals is especially evident when examining the corresponding cognitive deficits, especially in regard to memory [20]. Of the over two million elderly Americans experiencing food insecurity [21], a significant portion identify as a racial or ethnic minority, have less than a high school education, are Medicare eligible, and are obese [22], thereby indicating a unique and unprecedented vulnerability to food insecurity in already underserved populations. Additionally, women and female headed households may be unduly impacted by food insecurity as compared to male-headed households [23, 24].

### Food stress in animal models

Although food insecurity has not previously been examined in a laboratory animal model, previous research has shown that stress can directly influence food consumption in rodents with exposure to mild stress (i.e. handling by researchers) being directly related to mild decreases in food consumption and exposure to high degrees of stress (i.e. restraint stress) being directly related to temporary, but significant decreases in food consumption [25]. Moreover, this finding shows that rodent models are sensitive to stress in relation to eating behaviors, but not to the extent that would overshadow the impact of a food access model. Additionally, research has shown that rodents are sensitive to changes in diet and food preference [26] and are sensitive to food-cost [27, 28]. Taken together, these findings indicate that rodent models are appropriate for examining the influence of external stress and food availability on feeding behavior.

### Need for research

With an estimated 40 million people in the United States living in food-insecure households, understanding the association between food-related uncertainty and both mental and physical health conditions is critical [29]. However, studying food insecurity in humans is inherently challenging, due to the large number of correlated risk factors and environmental stressors. Despite the prevalence of food insecurity and the complications surrounding human research in this area, there is currently no laboratory animal model of food insecurity. This study deliberately and explicitly develops an animal model to mirror specific human experiences relevant to public health. This research is not intended to provide new solutions to the problem of food insecurity (e.g. increasing food availability and affordability), but rather, attempts to better understand the physiological and behavioral effects of food insecurity to highlight consequences of food insecurity, independent of other risk factors.

### The current study

Examining the consequences of inconsistent access to food as a predictor of poor health using an animal model could provide insight into the relationship between the persisting prevalence of food insecurity and chronic illness, independent of the complex clustering of risk factors seen in humans.

In a controlled laboratory environment, food insecurity can be isolated from other social, economic, and physical factors that impact health [30]. Examining links between food insecurity and negative health outcomes can inform policies by exemplifying the detrimental impact of social and economic inequality that allows for food insecurity to persist. This explorative study examines the relationship between food insecurity in older female mice and a range of behavioral outcomes, including food consumption, anxiety-related behaviors, active coping mechanisms, and memory.

## Method

### Animals

Aged female CD-1 mice, acquired from Charles River at PND23, were housed 3–5 mice per cage in 48 cm (L) x 27 cm (W) x 20 cm (H) polycarbonate cages with pine shavings bedding (7088 Teklad, Envigo) on a 12:12 hour light:dark schedule with the lights on at 7:00 am. All mice were housed 5/cage throughout life, but cage sizes differed by the time this study was conducted due to the fact that 15 mice passed due to natural causes. The temperature of the room was 21.1°C (SD = 0.50), with 49.5% humidity (SD = 2.5). Except during the food insecurity protocol, mice received *ad libitum* access to standard chow (3.1kcal/g, 18% energy from fat; https://insights.envigo.com/hubfs/resources/data-sheets/2018-datasheet-0915.pdf) All mice had access to water at all times. Throughout the duration of the study, mice were weighed twice a week. The Santa Clara University Institutional Animal Care and Use Committee (IACUC) approved this study. NIH guidelines for the care and use of laboratory animals were followed. Mice were euthanized using CO_2_, consistent with NIH guidelines.

### Food insecurity

Mice were assigned to food insecurity vs. control using alternating assignment of cages; there was no significant difference in weight between mice in the food insecurity and control conditions prior to the food insecurity exposure period (T-test: T = 0.20, p = 0.84). Mice exposed to food insecurity (8 cages, 31 mice) received unpredictable amounts of standard chow for four days each week, followed by *ad libitum* access to chow for three days, for four consecutive weeks (Table 1), beginning when mice were approximately 18 months old, generally considered the beginning of old age [31]. During each 4-day exposure period to food insecurity, mice in the food insecure group received food ranging between 50% and 150% of their baseline calorie consumption (50%, 75%, 125%, 150% of baseline), in an unpredictable pattern. Food was weighed daily during periods of food insecurity, and following each three-day recovery period, to assess consumption at the cage level. This pattern was intended to approximate the pattern of food insecurity in humans, where there may be sufficient food at the beginning of the month, followed by unpredictable access for the remainder. Control mice (8 cages, 34 mice) had *ad libitum* access to food throughout the study.

**Table 1:** Food insecurity protocol timeline.

	Standard chow received per cage (% of baseline^a^)				
Week of Protocol	Day 1	Day 2	Day 3	Day 4	Days 5–7
**1**	75%	150%	50%	125%	500%
**2**	125%	50%	150%	75%	500%
**3**	150%	75%	125%	50%	500%
**4**	50%	125%	75%	150%	500%

^a^Daily baseline consumption, (mean (SD)): 4.15 (0.53) g/mouse, 12.86 (1.63) kcal/mouse.

### High fat food consumption

Mice received high fat food (4.65kcal/g, 45% energy from fat; https://www.testdiet.com/cs/groups/lolweb/@testdiet/documents/web_content/mdrf/mdi2/~edisp/ducm04_026207.pdf) overnight for one day following one month of ad libitum access to standard chow after the food insecurity protocol. Food consumption over 24 hours was measured at the cage level. Within each cage, average kcal consumed per mouse was calculated. Data were analyzed at the cage-level.

### Behavioral testing

Behavioral testing occurred both before and after the food insecurity protocol (Table 2). During behavioral testing, all mice were given *ad libitum* food. All mice underwent the same schedule of tests; within each test the order in which mice were evaluated was counterbalanced with regard to the experimental group. Tests were video recorded and scored by well-trained observers without reference to which mice were part of the food insecurity exposure condition and spot-checked for consistency by a second researcher.

**Table 2:** Behavioral testing schedule.

Behavioral Test	Mouse Age (days)
Light/Dark Box	357
Light/Dark Box + Restraint	377
Elevated Plus Maze	393
High Fat Food Consumption	421
Forced Swim Test	447
Object Recognition	512, 513
**Food Insecurity**	**517–545**
Light/Dark Box	553
Forced Swim Test	559
Object Recognition	560, 561
Light/Dark Box + Restraint	567
High Fat Food Consumption	580
Elevated Plus Maze	597

### Forced swim test

Mice were placed in cylindrical containers with 15 cm of water at 24°C. The mice were filmed for six minutes, removed from the containers, dried, and returned to their cages. Behavior (active escaping behavior; i.e.—attempting to climb out of the water) was scored for the final four minutes of the test [32, 33]. Water was replaced for each mouse.

### Light-dark box

Behavior in the light-dark box (80 cm (L) x 40 cm (W) x 20cm (H) plexiglass box with a transparent (light) half and a black (dark) half) was video recorded for 5 minutes. Time spent in the light compartment was scored [34]. The test was repeated following 15 minutes of restraint stress by immobilization in disposable polyethylene conical restraints (DecapiCone, Braintree Scientific Inc., Braintree, Massachusetts) with a small hole at the tip to allow breathing.

### Elevated plus maze

The maze apparatus consisted of two orthogonal (112 x 10 cm) arms, one open and one enclosed by 40 cm high walls, elevated 50 cm above the ground. Behavior was video recorded for 5 minutes. Time spent on open arms was scored as exploratory behavior [35], using a scoring program [36]. Five mice (one from the food insecurity condition and four from the control group) fell from the elevated plus maze and were excluded from the analysis.

### Object recognition

On day 1 (familiarization), mice were allowed to explore two identical objects in a 48 cm (L) x 27 cm (W) x 20 cm (H) polycarbonate cage for 10 min. After 24 hours, recognition tests were conducted, in which one of the objects was randomly replaced with a novel, different object of similar dimensions and materials. The selection and location of novel vs. familiar objects was counterbalanced across groups; the objects were green plastic building blocks (Duplo Mega Blocks) and a glass and metal salt shaker prior to the food insecurity protocol and a plastic lemon and small plastic paint can after the food insecurity protocol. Time spent investigating each object was scored over 10 min [37, 38]. The proportion of time spent investigating the novel object, of the total time spent investigating both objects, was calculated for the recognition test.

### Statistical analysis

All behavioral tasks were conducted before and after the food insecurity protocol. Data were analyzed using two-way repeated measures ANOVA, including the effect of time, group, and the interaction of group and time; the normality of the data was verified visually using quantile-quantile plots and residual vs. fitted plots, as well as the Shapiro-Wilk test. Elevated plus maze data were log-transformed to meet the normality assumptions of ANOVA. All statistical tests used a two-tailed alpha level of 0.05. We used the Bonferroni adjustment for post-hoc comparisons following ANOVA. Statistical analysis was conducted using Stata 15 (College Station, TX).

## Results

### Food consumption

**Fig 1:**
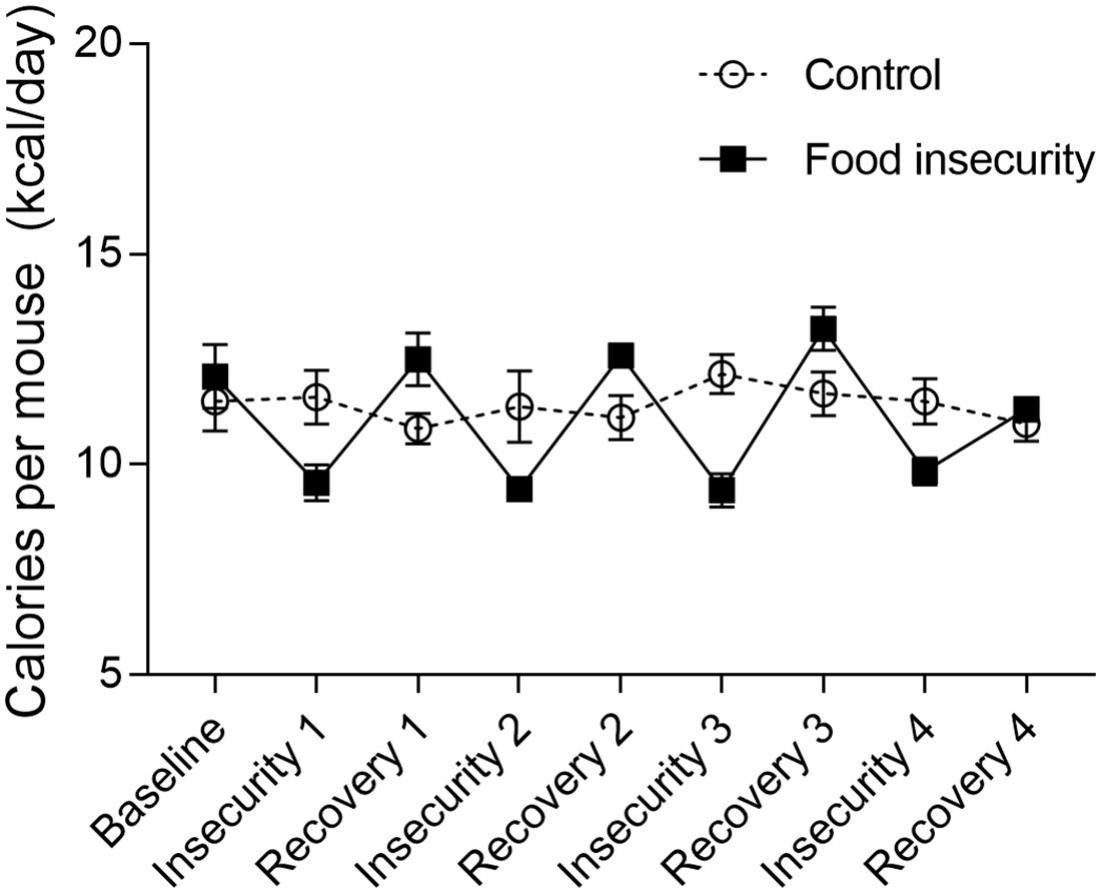
Food consumption during food insecurity protocol. Mice exposed to food insecurity received unpredictable, variable amounts of food for four days (Insecurity) followed by unlimited access to food for three days (Recovery). This pattern was repeated for four weeks. Control mice had unlimited access to food. Data show daily consumption per mouse (mean +/-SEM), by group. Baseline food consumption, prior to food insecurity exposure, did not differ by group (T-test: T = -0.58, df = 14, p = 0.57). During food insecurity and recovery, food consumption differed by group and time with mice exposed to food insecurity eating less than controls during food insecurity exposure and more during recovery (two-way ANOVA: group x time interaction: F_7,93_ = 10.95, P < 0.01).

Baseline food consumption did not differ between groups (T-test: T = -0.58, df = 14, P = 0.57). During food insecurity and recovery, food consumption differed significantly by group and time with mice exposed to food insecurity eating less than controls during periods of food insecurity and more during recovery (Fig 1; two-way ANOVA: group x time interaction: F_7,93_ = 10.95, P < 0.01). During food insecurity exposure periods, mice in the food insecure group ate 2.14 kcal less per mouse per day than controls, but left an average of 3.66 kcal per mouse per day uneaten. On exposure days when mice in the food insecurity condition were given more than 100% of baseline food consumption amounts, mice did not overeat to compensate for days they were fed less. During recovery periods, mice exposed to food insecurity ate 1.43 kcal per mouse per day more than controls.

After one month of ad libitum feeding following the food insecurity protocol, mice were provided with high (45%) fat food overnight. Both groups consumed more calories than when only chow was available. A group x time interaction was observed, such that cages of food insecure mice increased the calories consumed by 3.67 kcal per mouse from the pre-food insecurity to post-food insecurity timepoints, while control animals reduced their consumption over time by 2.21 kcal (two-way ANOVA, group x time interaction: F_1,14_ = 11.14, P < 0.01). Food insecure mice also consumed more calories from chow on the day following access to high fat food, compared to controls (two-way ANOVA: F_1,14_ = 20.86, P < 0.001).

Food insecurity did not affect the weight of mice. Mice in both groups gained weight over time (two-way ANOVA, main effect of time: F_2,126_ = 4.33, P = 0.015). There was no significant difference in weight by group (two-way ANOVA, main effect of group: F_1,126_ = 0.02, P = 0.89). Mice in the food insecurity condition did not lose weight during the food insecurity protocol, and there was no interaction found between group and time (two-way ANOVA, group x time interaction: F_2,126_ = 0.72, P = 0.49).

### Forced swim test

**Fig 2:**
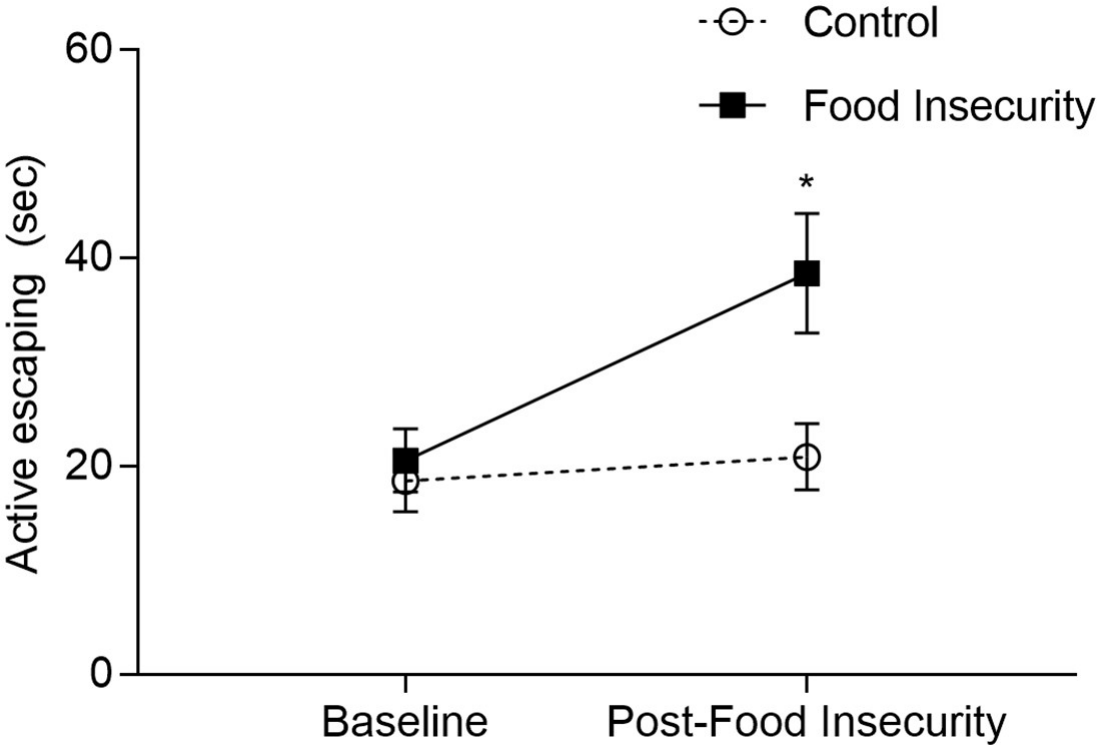
Effect of food insecurity on time spent actively coping in forced swim test (mean +/-SEM). An interaction was observed between group and time, such that mice exposed to food insecurity increased their active escaping behavior, while controls did not (repeated measures two-way ANOVA, group x time interaction: F_1,63_ = 5.40, P = 0.023). *P<0.05.

Food insecurity altered coping behaviors in the forced swim test (Fig 2). Both mice that experienced food insecurity and control mice did not differ significantly in the amount of escaping behavior prior to food insecurity exposure. Mice that underwent food insecurity showed a significant increase in active escaping behaviors following exposure as compared to those in the control group (repeated measures two-way ANOVA, group x time interaction: F_1,63_ = 5.40, P = 0.023). Active escaping behavior in the mice exposed to food insecurity increased by 18.0 seconds from the pre-exposure to the post-exposure test (T = 3.52, Bonferroni adjusted P = 0.002). However, mice in the control group only increased their active escaping behavior by 2.3 seconds between the two tests (T = 0.32, Bonferroni adjusted P = 1.0).

### Exploratory behavior

Exploratory behavior was not affected by food insecurity but did increase over time for both groups. Exploratory behavior in the light dark box increased in both groups over time under both non-stress conditions (repeated measures two-way ANOVA, main effect of time: F_1,63_ = 24.88, P < 0.001) and following restraint stress (repeated measures two-way ANOVA, main effect of time: F_1,63_ = 17.26, P < 0.001). Mice displayed less exploratory behavior when stressed (via restraint) than when tested under non-stress conditions.

In the elevated plus maze, time exploring the open arms increased over time (repeated measures two-way ANOVA, main effect of time: F_1,58_ = 4.15, P = 0.046). However, there was no significant effect of group, or the interaction between time and group.

### Memory

**Fig 3:**
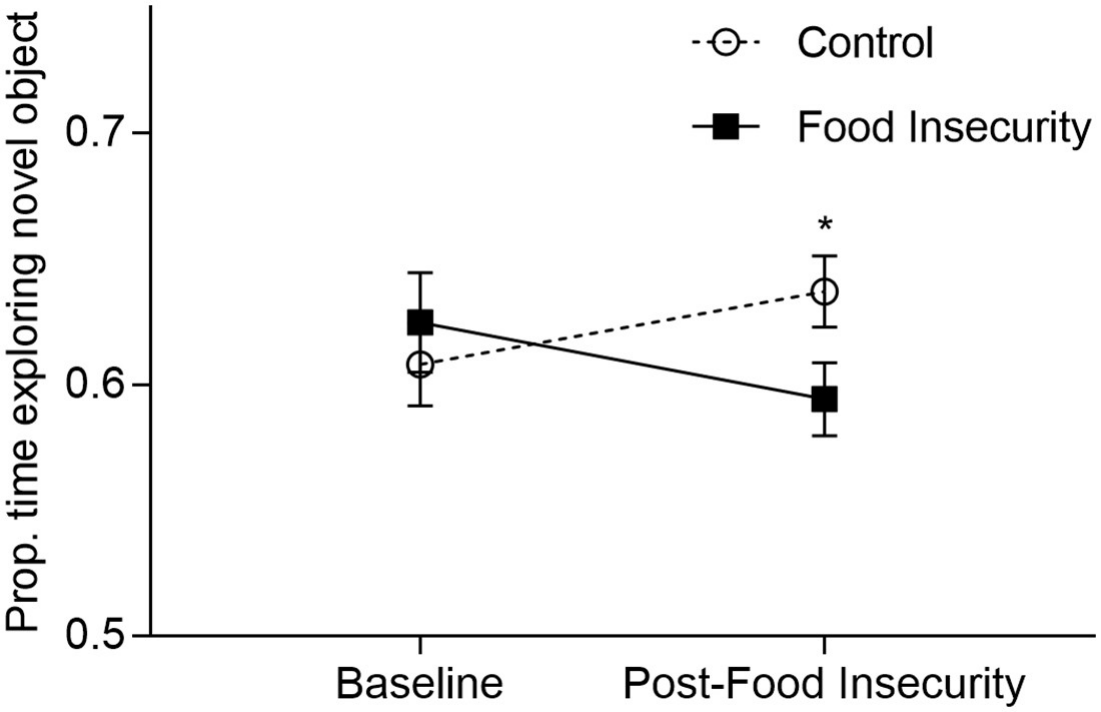
Effect of food insecurity on proportion of test time spent exploring novel object (mean +/-SEM). Mice were observed exploring unfamiliar objects before and after the food insecurity protocol. The proportion of time exploring the novel object increased among control mice (Δ +2.9%) but decreased among mice exposed to food insecurity (Δ -3.0%). (repeated measures two-way ANOVA, group x time interaction: F_1,62_ = 4.55, P = 0.037). *P<0.05.

Mice underwent object recognition testing before and after exposure to food insecurity (Fig 3). At baseline, the groups did not differ in the time spent exploring the novel objects. After the food insecurity protocol, mice exposed to food insecurity spent a lower proportion of time investigating the novel object relative to controls (repeated measures two-way ANOVA, group x time interaction: F_1,62_ = 4.55, P = 0.037). Control mice increased their proportion of time spent exploring the novel object, from 60.8% in test one to 63.7% in test two (+2.9%). Mice exposed to food insecurity decreased the proportion of time spent exploring the novel object from 62.5% in test one to 59.4% in test two (-3.0%). The decrease in novel object exploration time in mice exposed to food insecurity, compared to the increase in the control group, suggests that food insecurity may impair memory function in older mice.

## Discussion

### Implications of current findings

The results of this exploratory study begin to provide insight into the relationship between food insecurity, behavior, and health through a controlled animal model. Mice exposed to food insecurity altered their food consumption patterns, consumed more food with higher energy density after food insecurity, displayed an increase in active coping behaviors, and showed reduced learning/memory, compared to control mice following a 4-week exposure protocol. Exploratory behaviors and weight of the mice, however, were unaffected by the experience of food insecurity. Differences seen between study groups were not due to undernutrition or limited access to calories in the food insecurity group, as mice had sufficient access to calories each week, simply in an unpredictable pattern. Moreover, these results were not due to the acute consequences of food insecurity, but rather demonstrate the lasting effects of a history of food insecurity. Thus, these results suggest that the experience of food insecurity may have lasting consequences, even after the resumption of consistent access to food.

Food consumption in mice before and after exposure to food insecurity in the current study aligns with the pattern observed in binge-eating and obesity in humans [39, 40]. As seen in the current mouse model, mice exposed to food insecurity displayed behavior akin to binge-eating and consumed more food, higher in energy density during recovery periods when presented with unlimited food. Research suggests binge-eating in individuals experiencing food insecurity promotes fat storage leading to the group experiencing a higher prevalence of obesity when compared to food secure individuals [41]. Although the mice that experienced food insecurity in this study did increase consumption of both chow and high energy density food in a manner akin to binge-eating, there was not a corresponding increase in weight. Longer term exposure to high fat food may be necessary to observe changes in weight and fat storage. Additionally, research has shown that when not given access to *ad libitum* food, laboratory animals adapt both physiological and metabolically to the change [42]. This adaptation that occurs could explain the lack of differences in weight between mice exposed to food insecurity versus unexposed mice.

Results from the forced swim test show that mice that experienced food insecurity exhibited more active escaping behaviors than control mice. Although traditionally used to assess depression-like behaviors [43], the forced swim test has also been interpreted as measuring responses to an uncontrollable stressor, where escaping behaviors indicate a struggle to adapt or cope with stressful situations [44]. In the context of this study, the increased active escaping behavior in mice that experienced food insecurity suggests a relationship between food insecurity, adaptability, and ability to cope. Previous research has hypothesized that active coping in response to an inescapable stressor can be maladaptive [45], as attempts to escape the water are futile and further escaping behaviors only weary the mouse. Thus, individuals exposed to food insecurity may have a lower ability to adapt to stress and engage in coping behaviors that exceed rational behavior and result in more harm than good, however more research needs to be done.

Mice exposed to food insecurity had mildly worse memory compared to control mice, as shown by mice in the food insecurity exposure condition spending less time exploring the novel object in the object recognition test. These results align with a study of older adults where food insecurity was associated with deficiencies in memory and higher likelihood for cognitive impairment [46]. The difference between the memory of mice exposed to food insecurity and controls supports research that elderly individuals exposed to food insecurity show declines in cognitive functioning [20]. These findings suggest that the emotional and cognitive strain of unpredictable food access may hasten cognitive decline, making this issue especially salient among elderly individuals.

### Limitations

The timing and limited length of the food insecurity protocol used in this study (4 weeks) may have limited the severity of the stressor and resulting consequences. Behavioral tests were conducted following the conclusion of the food insecurity protocol, which may limit the strength of the observed effects. It is expected that behavioral effects of food insecurity may be stronger during food insecurity exposure, as the acute stress experience may be more salient than a history of stress exposure. Examination of exposure to food insecurity for longer periods of time or during critical developmental periods may yield additional or stronger results [47]. The consumption patterns of grazing versus bolus eating in the mice are unknown as well and could have affected the results. In addition, behavior was assessed only before and after food insecurity, so we are unable to identify behavioral changes during exposure to food insecurity.

This study did not assess the potential biological underpinnings of the behavioral findings. Future studies could examine differences in hormone levels or neuroendocrine pathways that may provide mechanisms for these findings. Only female mice were used in this study which limits the generalizability of the study while also allowing for greater insight into the specific impact of food insecurity on females. Additionally, the use of an animal model is inherently limited because it does not allow for a consideration of socially constructed gender roles, gender diversity, racism, and intersectionality.

### Current models and future research questions

Our model of food insecurity builds on previous work regarding unpredictable stress, food access, and food consumption. The unpredictability of our food insecurity protocol shares some similarities with chronic unpredictable stress protocol [48], but the behavioral consequences differ in some domains. For example, food insecurity was associated with increased active coping, whereas chronic unpredictable stress has been associated with passive coping (depressive phenotype) [49]. On the other hand, both paradigms are associated with impaired memory, relative to controls [50].

Similarly, animal models of bingeing behavior have found that limited access to high fat food is subsequently associated with an increase in bingeing [51] whereas intermittent exposure or consistent exposure is associated with decreases in bingeing behavior [52]. These models are similar to the findings in our study, where mice were observed to binge eat high fat food, especially following exposure to food insecurity. Overall, our study adds insight into the contribution of external stressors and food availability to binge eating behavior.

In future studies, food insecurity should be examined in relation to these models (e.g. similar outcomes, comparison of effects). Future research that directly explores biological underpinnings of the food insecurity paradigm, in comparison to chronic mild stress and models of bingeing behavior, would help to disentangle the consequences of unpredictability in food access versus other forms of stress. To further explore the food insecurity model, we plan to examine the effects of exposure to unpredictable food access during critical periods in development (e.g. pregnancy, postnatal, adolescence). In addition, the effects of food insecurity on developmentally appropriate behaviors should be assessed, to evaluate the immediate vs. lasting effects of food insecurity.

Furthermore, these findings present future research questions on the impact of food insecurity on the life course of an individual. Previous research has found that childhood and adolescent stress contributes to increased allostatic load in adulthood [53]. Thus, future research is needed to study how experiences of food insecurity in early life may have long term effects on health outcomes. Future studies should analyze the effects of food insecurity during sensitive periods, specifically that of early childhood, providing insight on the health impacts of food insecurity and related stress during development [54]. Additionally, the nature in which the mice responded to the forced swim test reinforces the interpretation of this task as assessing maladaptive coping mechanisms in addition to depression [44]. Further research ought to be done in order to determine the validity of the claims and the corresponding implications surrounding excessive, maladaptive coping behaviors in mice models. Finally, as the current study established a baseline for the behavioral outcomes associated with food insecurity in a mouse model, future research should examine the biological underpinnings of these behaviors by assessing biomarkers such as stress hormones throughout food insecurity manipulations and behavioral assessment.

## Conclusion

The development of this mouse research model allowed for the analysis of the lasting effect of food insecurity on the behavior of older female mice. This research documents behavioral changes in responses to stress, including food consumption, active coping behaviors, and memory. Overall, this research may inform the greater question of how food insecurity affects the physical and mental health of humans as well as provide a foundation for future animal models of food insecurity across developmental periods. Understanding the role that food insecurity and associated stress play in health and behavior may help inform policies and strategies to alleviate food insecurity in affected communities.
